# Comparing feedforward neural networks using independent component analysis on hidden units

**DOI:** 10.1371/journal.pone.0290435

**Published:** 2023-08-24

**Authors:** Seiya Satoh, Kenta Yamagishi, Tatsuji Takahashi

**Affiliations:** 1 School of Science and Engineering, Tokyo Denki University, Saitama, Japan; 2 Graduate School of Science and Engineering, Tokyo Denki University, Saitama, Japan; Hainan Normal University, CHINA

## Abstract

Neural networks are widely used for classification and regression tasks, but they do not always perform well, nor explicitly inform us of the rationale for their predictions. In this study we propose a novel method of comparing a pair of different feedforward neural networks, which draws on independent components obtained by independent component analysis (ICA) on the hidden layers of these networks. It can compare different feedforward neural networks even when they have different structures, as well as feedforward neural networks that learned partially different datasets, yielding insights into their functionality or performance. We evaluate the proposed method by conducting three experiments with feedforward neural networks that have one hidden layer, and verify whether a pair of feedforward neural networks can be compared by the proposed method when the numbers of hidden units in the layer are different, when the datasets are partially different, and when activation functions are different. The results show that similar independent components are extracted from two feedforward neural networks, even when the three circumstances above are different. Our experiments also reveal that mere comparison of weights or activations does not lead to identifying similar relationships. Through the extraction of independent components, the proposed method can assess whether the internal processing of one neural network resembles that of another. This approach has the potential to help understand the performance of neural networks.

## Introduction

Neural networks (NNs) have shown high performance in many tasks, such as image processing [1–3] and disease prediction [4, 5]. However, their high performance is not guaranteed, and when they performed poorly it is hard to determine what caused that. One of the reasons for the difficulty of diagnosing the causes is that NNs usually have numerous parameters and the representations are distributed, resulting in failure of revealing the grounds of their prediction result.

Explainable artificial intelligence research, which is the attempt to explicitly render the basis of the prediction results, has been actively conducted in recent years [6, 7]. For example, integrated gradients [8] and DeepLift (Deep Learning Important FeaTures) [9] are methods to obtain pixel importance based on gradients and give an explanation of where the model focused on to make their prediction. However, there are some shortcomings in the reliability of the explanation [10, 11]. For example in the cases of integrated gradients and DeepLift, it has been shown that it is possible to add a small perturbation to the input image so that the pixel importance significantly changes while the prediction results remain the same [10]. Also, it is difficult to explain the basis for highlighting the pixels as particularly important even when pixel importance can always be calculated well. For example, explaining whether the prediction was made on the basis of pixel color or shape is difficult.

In this study we propose a novel method which performs independent component analysis (ICA) on the outputs of hidden units of feedforward neural networks (FNNs), and compares those networks based on the obtained independent components (ICs). This method can be used even for comparing FNNs with different structures. The proposed method also can compare two FNNs that solve different tasks. These advantages can lead to useful findings. Despite numerous recent proposals for interpreting the internal representations of neural networks [12–14], to the best of our knowledge, no existing method applies ICA to analyze FNNs’ hidden layers. In our experiments we use FNNs with one hidden layer, and verify whether FNNs can be compared by the proposed method when the number of hidden units of FNNs is different, when the data is partially different, and when the activation function is different. Additionally, we demonstrate that a mere comparison of weights or activations does not lead to the discovery of similar relationships.

## FNNs and their properties

Here, we focus on FNNs with one hidden layer. Given the input ***x***^*μ*^, the output of the *j*th hidden unit is

$$\begin{array}{c} h_{j}^{\mu }=g\left({w_{j}}^{\text{T}}{\tilde{x}}^{\mu }\right) \end{array}$$ (1)

where ${\tilde{x}}^{\mu }={\left(1,{x^{\mu }}^{\text{T}}\right)}^{\text{T}}$, $x^{\mu }={\left(x_{1}^{\mu },\ldots ,x_{K}^{\mu }\right)}^{\text{T}}$, *K* is the number of input variables, $w_{j}={\left(w_{j,0},\ldots ,w_{j,K}\right)}^{\text{T}}$ are weights between the input layer and the *j*th hidden unit, and *g* is the activation function. Here we use either the logistic sigmoid function or the softplus function (a smooth version of rectified linear unit function) [15] as the activation function of the hidden layer. We use an identity function as the activation functions of the input and output layers. Given the input ***x***^*μ*^, the output of the *i*th output unit is

$$\begin{array}{c} f_{i}^{\mu }={v_{i}}^{\text{T}}h^{\mu } \end{array}$$ (2)

where $h^{\mu }={\left(1,h_{1}^{\mu },\ldots ,h_{J}^{\mu }\right)}^{\text{T}},$ *J* is the number of hidden units, and $v_{i}={\left(v_{i,0},\ldots ,v_{i,J}\right)}^{\text{T}}$ are weights between the hidden layer and the *i*th output unit. The parameters of an FNN with *J* hidden units are $\theta_{J}={\left({w_{1}}^{\text{T}},\ldots ,{w_{J}}^{\text{T}},{v_{1}}^{\text{T}},\ldots ,{v_{I}}^{\text{T}}\right)}^{\text{T}}$.

As the objective function we use the mean squared error (MSE). Given a dataset *D* = {(***x***^*μ*^, ***y***^*μ*^)|*μ* ∈ {1, …, *N*}}, the MSE is the following:

$$\begin{array}{c} MSE=\frac{1}{IN}\sum_{\mu =1}^{N}\sum_{i=1}^{I}(f_{i}^{\mu }-y_{i}^{\mu }{)}^{2} \end{array}$$ (3)

where $y^{\mu }={\left(y_{1}^{\mu },\ldots ,y_{I}^{\mu }\right)}^{\text{T}}$, *I* is the number of output units, and *N* is the number of data points.

The solutions of an FNN are usually non-unique and dependent on initial parameters due to the properties of the FNN. For example, the input-output function of an FNN remains unchanged when the order of hidden units is altered. In addition, some activation functions have symmetry and the logistic sigmoid function used in our experiments has the following symmetry:

$$\begin{array}{c} \sigma (x)=1-\sigma (-x). \end{array}$$ (4)

Furthermore, one property of FNNs is reducibility [16, 17]. For example, consider the optimal solution of an FNN with *J* hidden units ${\hat{\theta }}_{J}=({{\hat{w}}_{1}}^{\text{T}},\ldots ,{{\hat{w}}_{J}}^{\text{T}},{\hat{v}}_{1,0},\ldots ,{\hat{v}}_{1,J},{\hat{v}}_{2,0},\ldots ,{\hat{v}}_{2,J},\ldots ,{\hat{v}}_{I,0},\ldots ,{\hat{v}}_{I,J}{)}^{\text{T}}$ and the following region:

$$\begin{array}{c} \left\{\theta_{J+1}\right|w_{j}={\hat{w}}_{j},\;w_{J+1}={\left(a,0,\ldots ,0\right)}^{\text{T}},v_{i,j^{\prime }}={\hat{v}}_{i,j^{\prime }},v_{i,J+1}=0,j\in \left\{1,\ldots ,J\right\},\\ \;j^{\prime }\in \left\{0,\ldots ,J\right\},i\in \left\{1,\ldots ,I\right\}\}, \end{array}$$ (5)

where ***θ***_*J*+1_ is an FNN with *J* + 1 hidden units and *a* is a scalar variable. In this case, the input-output function of an FNN on the region is the same as that of the optimal solution ${\hat{\theta }}_{J}$ regardless of the value of *a*, and the gradient is zero. Therefore, an FNN with *J* + 1 hidden units on the region is reducible to the optimal solution. For the details of reducibility of FNNs, see [16, 17]. The characteristic of reducibility indicates that even when the weight values differ, the input-output relationship can remain consistent. As such, a simple comparison of FNNs’ weights is insufficient to establish similarities in their input-output relationships. The experimental findings discussed later confirm this observation.

## A method to compare FNNs

**Fig 1:**
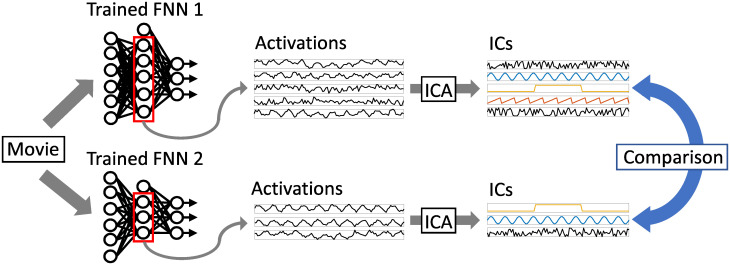
An illustration of our method to compare FNNs.

It is very difficult to compare FNNs to each other due to their properties, such as the ones mentioned in the previous section. In this paper we propose a method that compare FNNs based on independent components (ICs) obtained by independent component analysis (ICA). Algorithm 1 shows the procedure of the proposed method and Fig 1 shows an illustration of the proposed method.

**Algorithm 1** Our method to compare FNNs

1: Let two trained FNNs be FNN 1 and FNN 2

2: Input a video to FNN 1, perform ICA on the activations (outputs) of the hidden units of FNN 1, and let the obtained ICs be denoted by $p_{1},p_{2},\ldots ,p_{J_{1}}$ where *J*_1_ is the number of the hidden units of FNN 1.

3: Input a video to FNN 2, perform ICA on the activations of the hidden units of FNN 2, and let the obtained ICs be denoted by $q_{1},q_{2},\ldots ,q_{J_{2}}$ where *J*_2_ is the number of the hidden units of FNN 2.

4: Calculate dissimilarities between $p_{1},p_{2},\ldots ,p_{J_{1}}$ and $q_{1},q_{2},\ldots ,q_{J_{2}}$.

In Steps 2 and 3 of Algorithm 1 we use ICA [18]. Among several ICA methods, we have chosen to use second-order blind identification (SOBI) [19, 20] (source code available at https://github.com/aludnam/MATLAB/blob/master/sobi/sobi.m). Videos used in Steps 2 and 3 need to be selected appropriately depending on what the FNNs have learned. The simplest way to do this is to use the images (input signals) of the dataset used to train FNN 1 (or FNN 2) as a video. We use this straightforward approach in our experiments.

In Step 4 the dissimilarity of the obtained ICs is calculated. Here, we use the following function that uses the Euclidean distance:

$$d(p,q)=\text{min}\{d_{E}\left(p,q\right),d_{E}\left(p,-q\right)\},$$ (6)

$$d_{E}\left(p,q\right)\equiv \left|\right|\tilde{p}-\tilde{q}\left|\right|,$$ (7)

where ***p*** and ***q*** are ICs, the length of ***p*** is the same as that of ***q***, and $\tilde{p}$ and $\tilde{q}$ are vectors normalized so that the means of ***p*** and ***q*** are 0 and the variances are 1. The reason for using *d*_*E*_(***p***, ***q***) and *d*_*E*_(***p***, −***q***) in Eq (6) is that the significance of an IC does not change even when the sign of the IC is inverted.

## Experiments

We conducted three experiments to evaluate the proposed method. In the first experiment (Experiment 1) we verified whether FNNs could be compared to each other by the proposed method when the numbers of hidden units of two FNNs were different. In the next experiment (Experiment 2) we verified whether FNNs that learned partially different teacher signals could also be compared. In the final experiment (Experiment 3) we verified whether FNNs could be compared when the activation functions of two FNNs were different. Across all three experiments, we also demonstrated that simply comparing weight values or activations between two FNNs did not necessarily reveal similarities between them. The comparison of weights was performed by calculating the Euclidean distance between the weights from the input layer to each hidden unit. The comparison of activations was done similarly to the comparison of ICs in our method, utilizing the dissimilarity measure given by Eq (6).

We generated datasets using Disentanglement testing Sprites dataset (dSprites) [21]. We used a computer with an Intel(R) Core(TM) i9-9900X CPU @ 3.50GHz and an NVIDIA GeForce RTX 2080 SUPER with MATLAB R2018b. We normalized input signals $x_{1}^{\mu },\ldots ,x_{K}^{\mu }$ and teacher signals $y_{1}^{\mu },\ldots ,y_{I}^{\mu }$ as follows:

$${\tilde{x}}_{k}^{\mu }\leftarrow \frac{x_{k}^{\mu }-{\text{min}}_{\mu }\left(x_{k}^{\mu }\right)}{{\text{max}}_{\mu }\left(x_{k}^{\mu }\right)-{\text{min}}_{\mu }\left(x_{k}^{\mu }\right)},$$ (8)

$${\tilde{y}}_{i}^{\mu }\leftarrow \frac{y_{i}^{\mu }-y_{i}^{\text{mean}}}{y_{i}^{\text{std}}},$$ (9)

where

$$y_{i}^{\text{mean}}=\frac{1}{N}\sum_{\mu =1}^{N}y_{i}^{\mu },$$ (10)

$$y_{i}^{\text{std}}=\sqrt{\frac{1}{N}\sum_{\mu =1}^{N}{\left(y_{i}^{\mu }-y_{i}^{\text{mean}}\right)}^{2}},$$ (11)

*μ* ∈ {1, …, *N*}, *k* ∈ {1, …, *K*}, and *i* ∈ {1, …, *I*}. We used FNNs whose outputs are written in Eq (2). As a learning method we used scaled conjugate gradient obtained from the Netlab toolbox (available at https://jp.mathworks.com/matlabcentral/fileexchange/2654-netlab) [22]. For each learning trial we set the maximum number of epochs to 1,000.

### Experiment 1: FNNs with different numbers of hidden units

**Fig 2:**
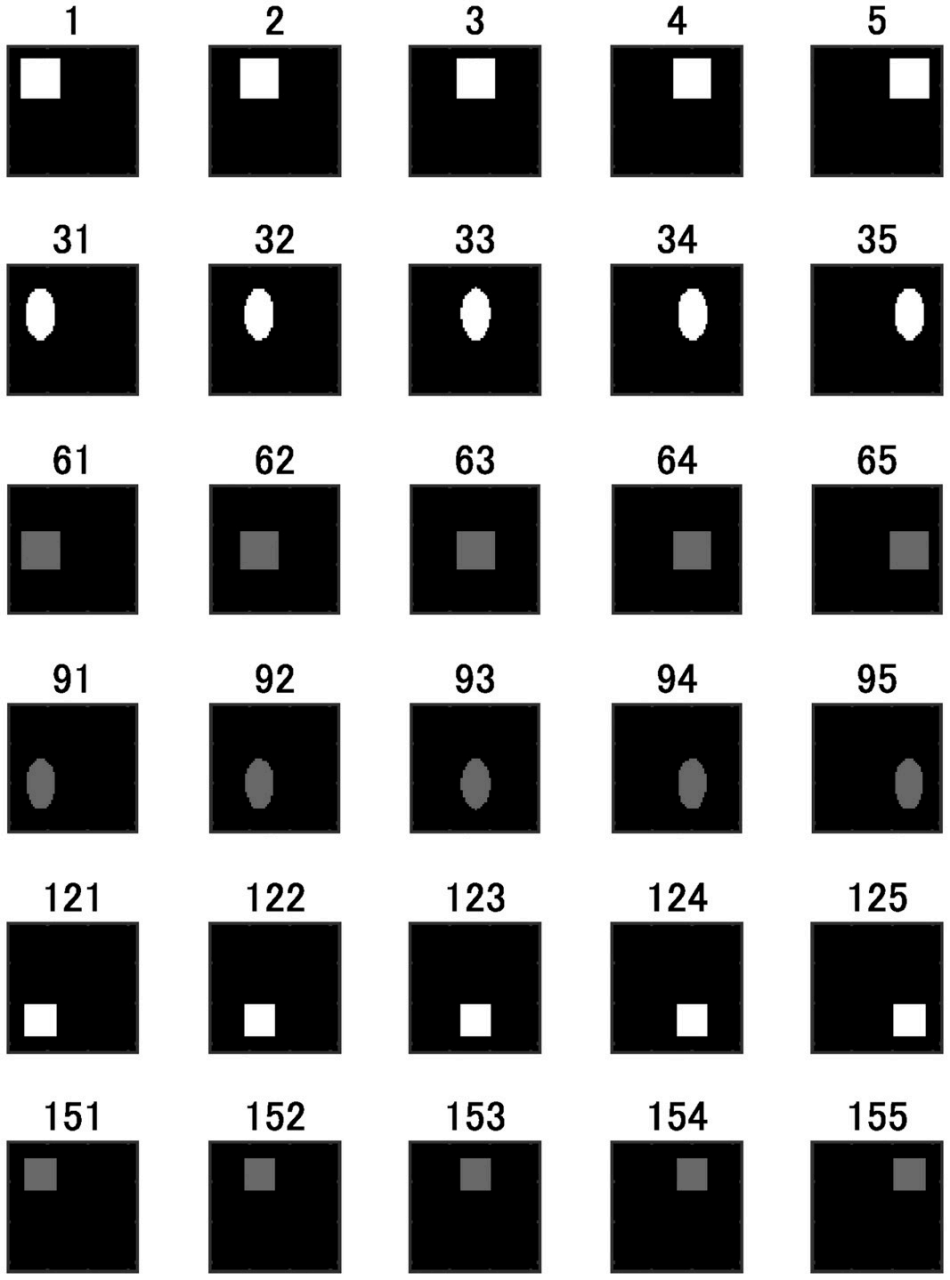
Input images. The number above each image represents the data number.

In Experiment 1 we verified whether two FNNs could be compared by the proposed method when the numbers of the hidden units of these FNNs were different. We used the logistic sigmoid function as the activation function of the hidden layers. Fig 2 shows a part of the input signals (images) of the dataset. We used the size (0.7 or 1), shape (square or ellipse), and color (white or dim gray) of the figure in an input signal as the teacher signal. We set the number of pairs of an input signal and teacher signal to 200 (= 2 (size) × 2 (shape) × 2 (color) × 25(position)). We call this dataset 1. Table 1 summarizes all datasets used in our experiments.

**Table 1:** Datasets used in the experiments.

		Dataset 1	Dataset 2
**Input signals**	**Size**	2 types (0.7 or 1)	
	**Shape**	2 types (square or ellipse)	
	**Color**	2 types (white or dim gray)	
	**Position**	25 types	
**Teacher signals**	**Size**	✓	
	**Shape**	✓	✓
	**Color**	✓	
	**Position**		✓

**Fig 3:**
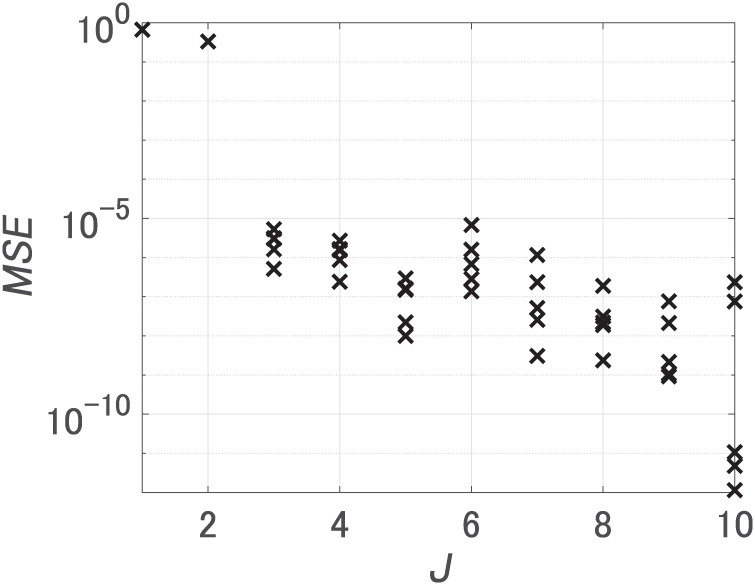
MSEs for dataset 1. *J* is the number of hidden units.

First, we set the number of hidden units *J* from 1 to 10, and performed 5 trials for each *J* changing initial weights. Fig 3 shows MSEs after learning. When *J* was 3 or more, the minimum value of MSEs was 10^−5^. Notably, an increase in the number of hidden units from 2 to 3 led to a significant decrease in the MSE, implying a necessity for at least 3 hidden units. We call the FNN with the smallest MSE at *J* = 3 FNN 1 and the FNN with the smallest MSE at *J* = 10 FNN 2. Table 2 encompasses all FNNs compared using our method.

**Table 2:** FNNs.

	Number of hidden units	Activation function	Dataset
**FNN 1**	3	Logistic sigmoid	Dataset 1
**FNN 2**	10	Logistic sigmoid	Dataset 1
**FNN 3**	10	Logistic sigmoid	Dataset 2
**FNN 4**	3	Softplus	Dataset 1

**Fig 4:**
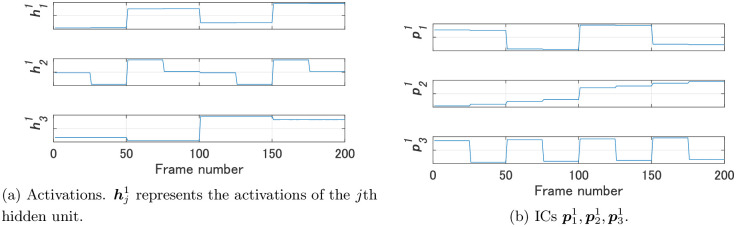
Activations of the hidden units of FNN 1 and their ICs for Experiment 1.

**Fig 5:**
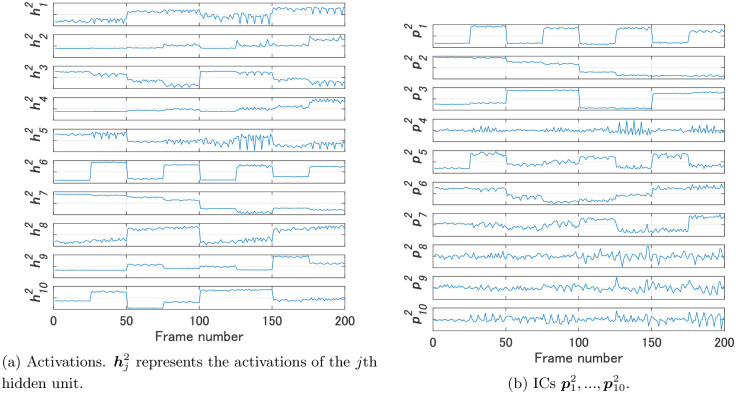
Activations of the hidden units of FNN 2 and their ICs for Experiment 1.

We compared FNN 1 and FNN 2 using the proposed method. As the video for Steps 2 and 3 of Algorithm 1 we used a video in which the images of dataset 1 were arranged in the order of data numbers. Fig 4 shows the activations $h_{1}^{1},h_{2}^{1},h_{3}^{1}$ of the hidden units of FNN 1 and their ICs $p_{1}^{1},p_{2}^{1},p_{3}^{1}$; Fig 5 shows the activations $h_{1}^{2},\ldots ,h_{10}^{2}$ of the hidden units of FNN 2 and their ICs $p_{1}^{2},\ldots ,p_{10}^{2}$. Note that the magnitude of the values of an IC shown in Figs 4b and 5b has no meaning, since each IC was normalized before calculating dissimilarities.

**Fig 6:**
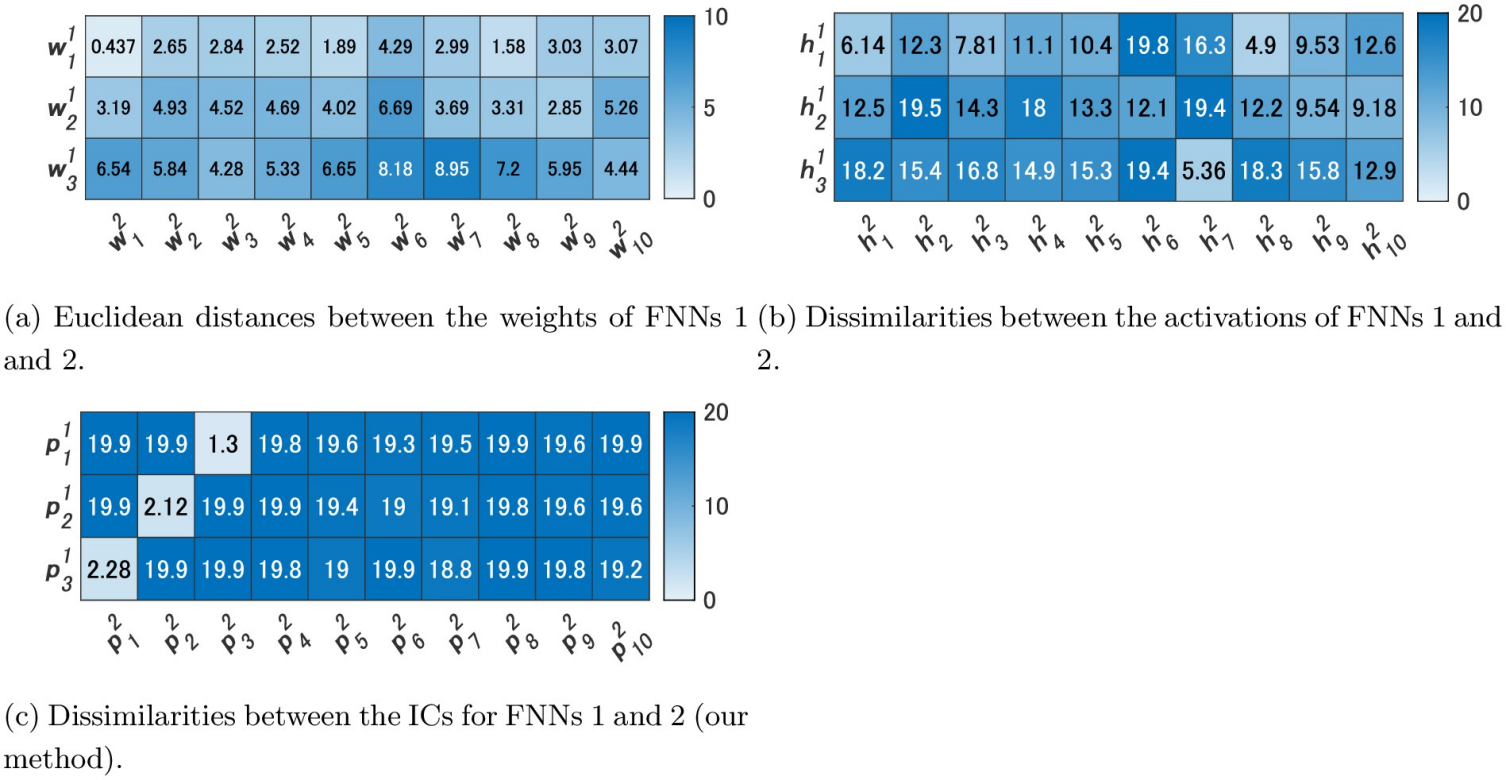
Heatmaps of the Euclidean distances and dissimilarities for Experiment 1.

Fig 6c shows the dissimilarities between the ICs of FNNs 1 and 2. In addition, Fig 6a shows the Euclidean distances between the weights $w_{1}^{1},w_{2}^{1},w_{3}^{1}$ of FNN 1 and those $w_{1}^{2},\ldots ,w_{10}^{2}$ of FNN 2. Meanwhile, Fig 6b shows the dissimilarities between the activations of FNNs 1 and 2. The values in Fig 6c are the values of dissimilarities. For example, the value in the first column of the third row is the value of $d(p_{3}^{1},p_{1}^{2})$, which is 2.28. As shown in Fig 6b and 6c, all the dissimilarity values were greater than 5 except for $d(h_{1}^{1},h_{8}^{2})$ in the comparisons of activations. Alternatively, $d(p_{1}^{1},p_{3}^{2})$, $d(p_{2}^{1},p_{2}^{2})$, and $d(p_{3}^{1},p_{1}^{2})$ were less than 5 in the comparisons of ICs. Activations of FNN 1 were not similar to those of FNN 2 in most cases, but three ICs of FNN 1 were similar to three ICs of FNN 2, each of which is considered an IC that contributes to size, color, and shape recognition.

As depicted in Fig 6a, when merely comparing weights, the weights $w_{1}^{2}$ of FNN 2 were most similar to the weights $w_{1}^{1}$ of FNN 1, with the Euclidean distance being 0.437. However, the smallest Euclidean distance to the weights $w_{2}^{1}$ was 2.85, which was 6.5 times the Euclidean distance between $w_{1}^{1}$ and $w_{1}^{2}$. Similarly, the smallest Euclidean distance to $w_{3}^{1}$ was 4.28, which was 9.8 times larger than the Euclidean distance between $w_{1}^{1}$ and $w_{1}^{2}$. Consequently, we were unable to find weights similar to $w_{2}^{1}$ and $w_{3}^{1}$.

From the above we consider that similar ICs could be extracted in two FNNs with different numbers of hidden units using our method. In contrast, a simple comparison of the weight values or activations between the two FNNs did not yield any significant findings of similar relationships.

### Experiment 2: Partially different teacher signals

In Experiment 2 we verified whether two FNNs that learned partially different teacher signals could also be compared by our method. We used an FNN that learned a different dataset (dataset 2) than dataset 1 and compared that FNN and another FNN that learned dataset 1. Specifically, the input images of dataset 2 were the same as those of dataset 1, and the teacher signal was changed to the shape (square, or ellipse) and the position of the figure for learning. Therefore, only the shape is included in the teacher signals of both dataset 1 and dataset 2. We used the same activation functions as in Experiment 1.

**Fig 7:**
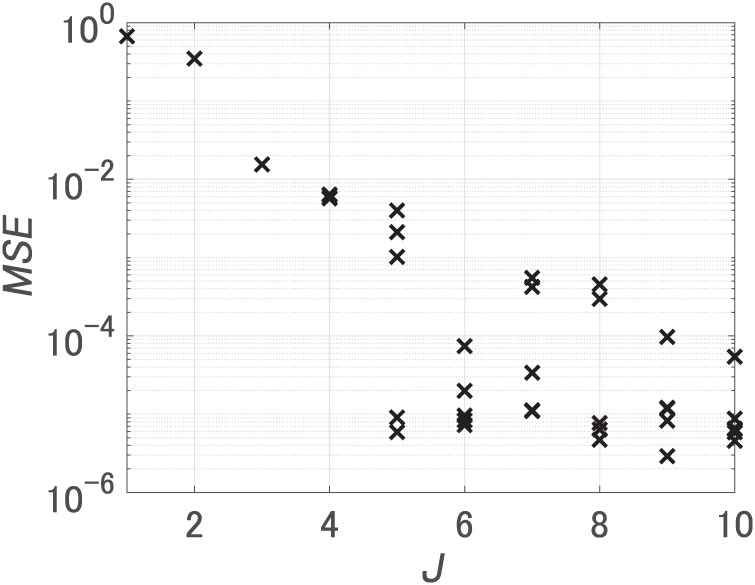
MSEs for dataset 2. *J* is the number of hidden units.

First, we set the number of hidden units *J* from 1 to 10, and performed 5 trials to learn dataset 2 for each *J* changing initial weights. Fig 7 shows MSEs after learning. We used the FNN with the smallest MSE at *J* = 10 as FNN 3.

**Fig 8:**
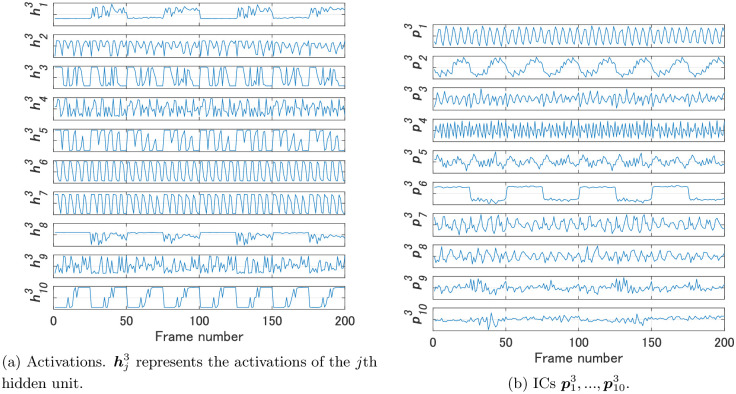
Activations of the hidden units of FNN 3 and their ICs for Experiment 2.

**Fig 9:**
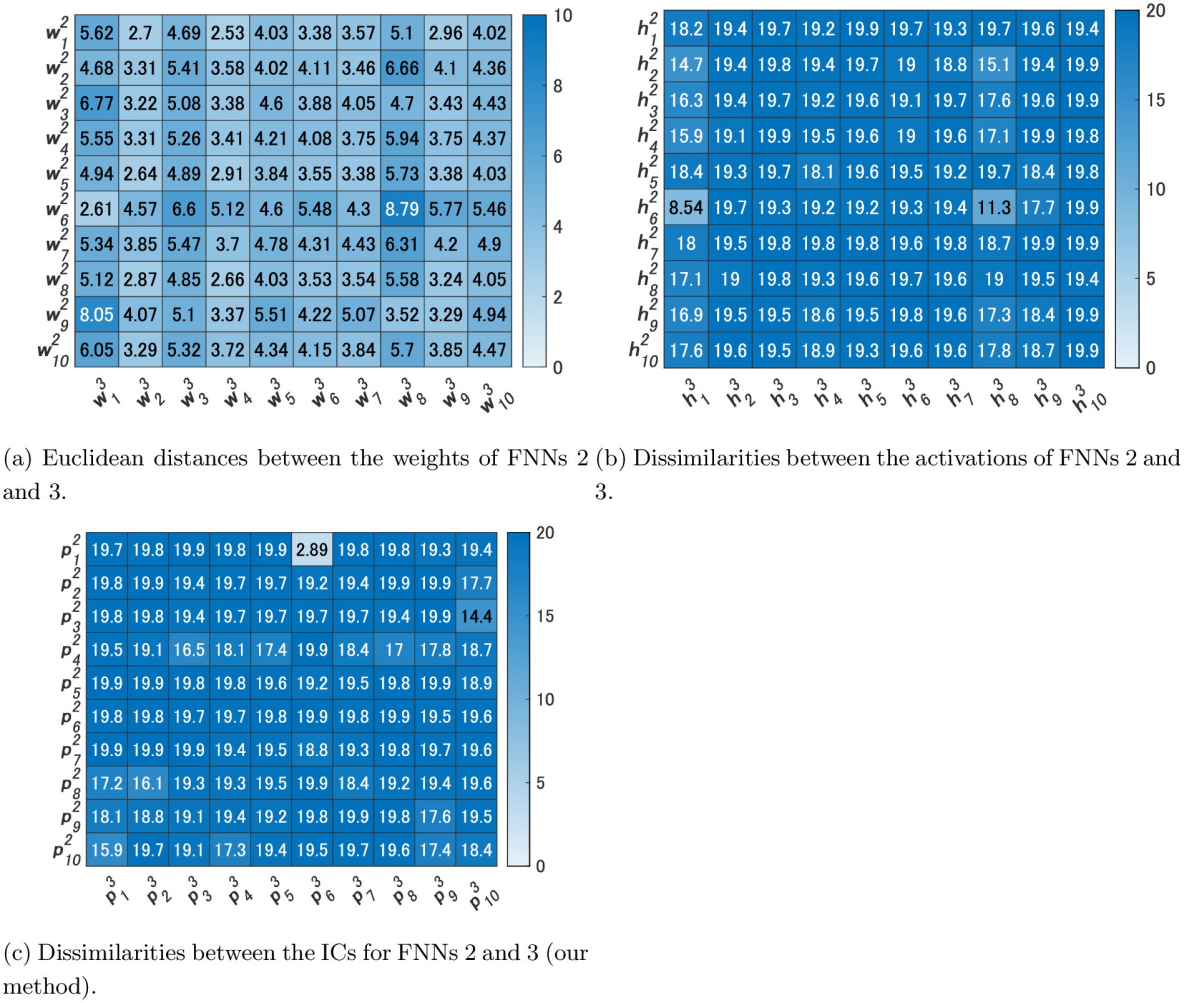
Heatmaps of the Euclidean distances and dissimilarities for Experiment 2.

We compared FNN 2, which we used in Experiment 1, and FNN 3 by our method. We used the same video used in Experiment 1 for Steps 2 and 3 of Algorithm 1. Fig 8 shows the activations $h_{1}^{3},\ldots ,h_{10}^{3}$ of the hidden units of FNN 3 and their ICs $p_{1}^{3},\ldots ,p_{10}^{3}$. Fig 9c shows the dissimilarities between the ICs of FNNs 2 and 3. In addition, Fig 9a shows the Euclidean distances between the weights $w_{1}^{2},\ldots ,w_{10}^{2}$ of FNN 2 and those $w_{1}^{3},\ldots ,w_{10}^{3}$ of FNN 3, and Fig 9b shows the dissimilarities between the activations of FNNs 2 and 3. As shown in Fig 9b and 9c, while all the values were greater than 5 in the comparisons of the activations, $d(p_{5}^{2},p_{6}^{3})$ was a small value in the comparisons of the ICs. These ICs are considered to contribute to shape recognition.

When simply comparing weights as shown in Fig 9a, the smallest Euclidean distance was between weights $w_{1}^{2}$ of FNN 2 and $w_{4}^{3}$ of FNN 3. However, the value was not below 0.5 as in Experiment 1, but was 2.53. It was found that the distribution of values did not clearly distinguish between those that are similar and those that are not, as in the comparison of ICs in our method.

From the above, we consider that our method could extract similar ICs from two FNNs that had learned from partially different teacher signals. However, simple weight or activation comparison between the two FNNs did not result in meaningful discovery of similar relationships.

### Experiment 3: Different activation functions

In Experiment 3 we verified whether two FNNs could be compared by the proposed method when the activation functions were different. We used the logistic sigmoid and softplus functions as the activation functions of the hidden layers. We used FNN 2 as an FNN with the logistic sigmoid function, which we used in Experiments 1 and 2.

**Fig 10:**
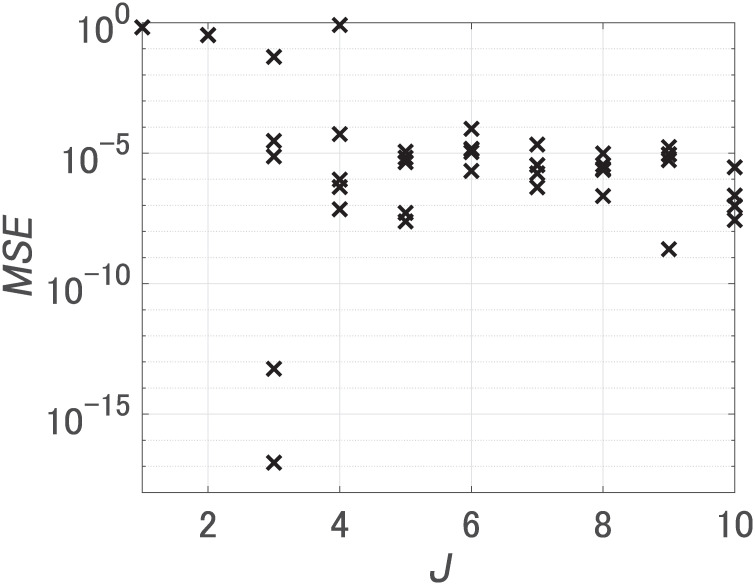
MSEs for dataset 1 (FNNs with softmax function). *J* is the number of hidden units.

In order to prepare a trained FNN with the softplus function, we first set the number of hidden units *J* from 1 to 10 and performed 5 trials to learn dataset 1 for each *J* changing initial weights. Fig 10 shows MSEs after learning. We used the FNN with the smallest MSE at *J* = 3 as FNN 4.

**Fig 11:**
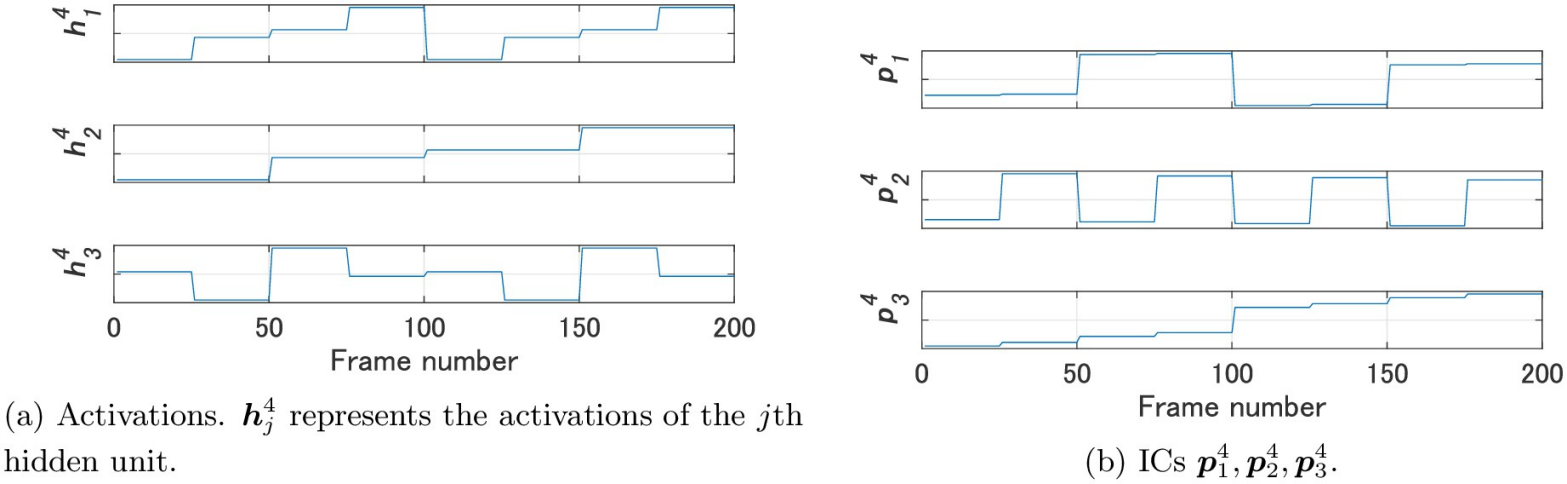
Activations of the hidden units of FNN 4 and their ICs for Experiment 3.

**Fig 12:**
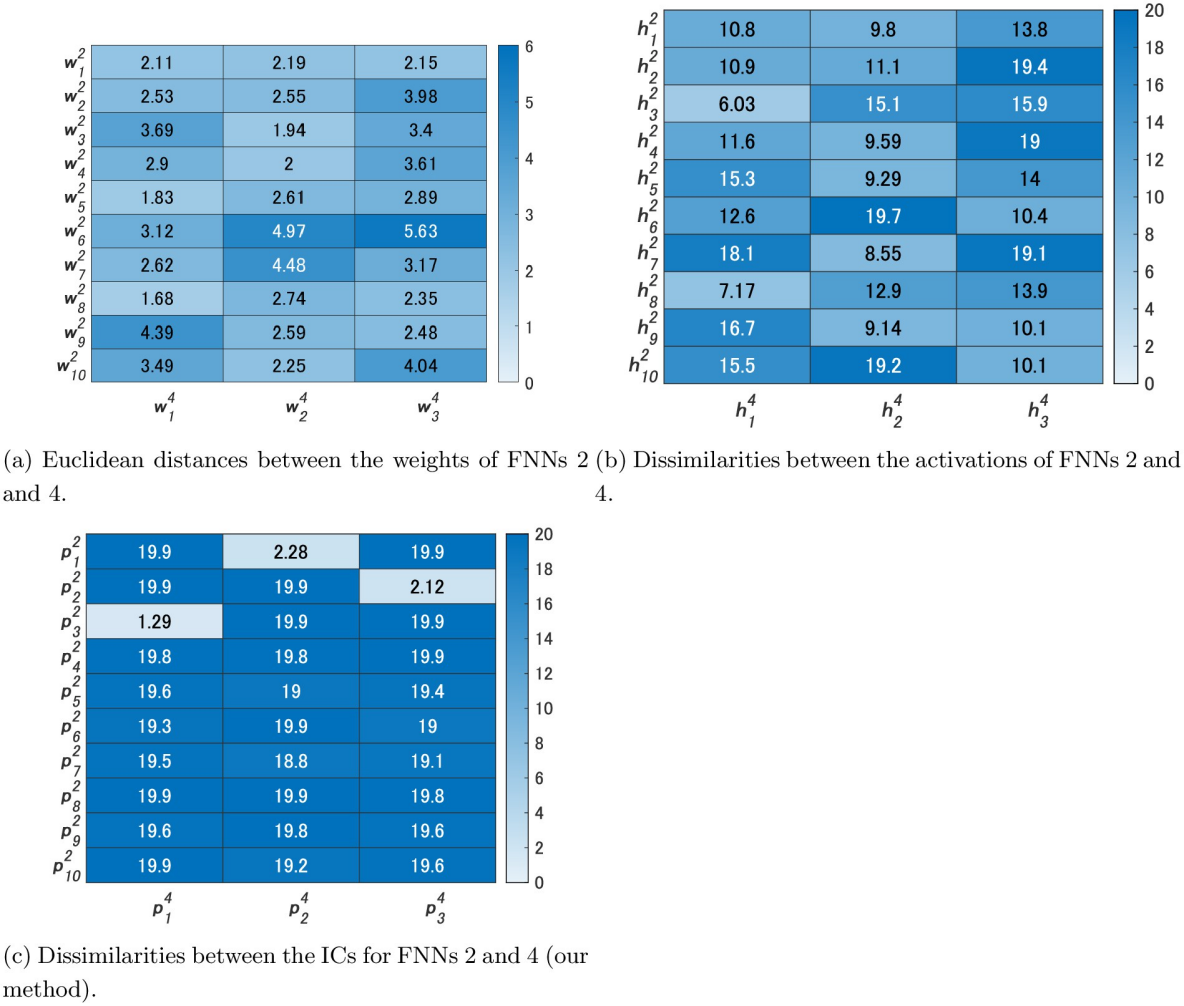
Heatmaps of the Euclidean distances and dissimilarities for Experiment 3.

We compared FNN 2 and FNN 4 by our method. We used the same video used in Experiments 1 and 2 as the video for Steps 2 and 3 of Algorithm 1. Fig 11 shows the activations $h_{1}^{4},h_{2}^{4},h_{3}^{4}$ of the hidden units of FNN 4 and their ICs $p_{1}^{4},p_{2}^{4},p_{3}^{4}$. Fig 12c shows the dissimilarities between the ICs of FNNs 2 and 4. In addition, Fig 12a shows the Euclidean distances between the weights $w_{1}^{2},\ldots ,w_{10}^{2}$ of FNN 2 and those $w_{1}^{4},w_{2}^{4},w_{3}^{4}$ of FNN 4, and Fig 12b shows the dissimilarities between the activations of FNNs 2 and 4. As shown in Fig 12b and 12c, while all the values were greater than 5 in the comparisons of the activations, the values $d(p_{3}^{2},p_{1}^{4})$, $d(p_{1}^{2},p_{2}^{4})$, and $d(p_{2}^{2},p_{3}^{4})$ were less than 5 in the comparisons of ICs. Activations of FNN 2 were not similar to those of FNN 4, but three ICs of FNN 2 were similar to three ICs of FNN 4, each of which was considered an IC that contributes to size, color, and shape recognition, as in Experiment 1.

As shown in Fig 12a, simply comparing weights did not lead to the meaningful discovery of similar relationships. similar ICs were extracted in two FNNs with different activation functions.

## Conclusions

In this paper we proposed a novel method of comparing FNNs based on the ICs obtained by performing ICA on the hidden layers of the FNNs. In our three experiments we showed that similar ICs were extracted from two FNNs, even when the numbers of hidden units were different; that similar ICs were extracted from two FNNs, even when the teacher signals were partially different; and that similar ICs were extracted from two FNNs, even when the activation functions were different. Our findings also revealed that direct comparisons of weights or activations did not uncover meaningful similarities. Note that we used only FNNs with a single hidden layer, utilizing either the logistic sigmoid function or the softplus function as the activation function. Also note that we used only SOBI as ICA and datasets generated using dSprites.

In the future, we will further investigate the availability of our method by conducting more complex experiments, such as experiments with FNNs that have two or more hidden layers, experiments with more complex data, and experiments with nonlinear ICA.
